# Genotypic, Environmental, and Processing Effects on Phenolic Content and Antioxidant Activity in Barley and Wheat

**DOI:** 10.3390/plants14111664

**Published:** 2025-05-30

**Authors:** Gordana Šimić, Alojzije Lalić, Daniela Horvat, Marija Viljevac Vuletić, Krešimir Dvojković, Marko Jukić, Jasmina Lukinac, Ivan Abičić

**Affiliations:** 1 Agricultural Institute Osijek, Južno Predgrađe 17, 31000 Osijek, Croatia; gordana.simic@poljinos.hr (G.Š.); alojzije.lalic@poljinos.hr (A.L.); daniela.horvat@poljinos.hr (D.H.); marija.viljevac@poljinos.hr (M.V.V.); kresimir.dvojkovic@poljinos.hr (K.D.); ivan.abicic@poljinos.hr (I.A.); 2 Faculty of Food Technology Osijek, Josip Juraj Strossmayer University of Osijek, F. Kuhača 18, 31000 Osijek, Croatia; marko.jukic@ptfos.hr

**Keywords:** barley, wheat, phenolic content, antioxidant activity, growing year, malting

## Abstract

Phenolic compounds are increasingly valued for their contribution to the antioxidant capacity and nutritional characteristics of cereals. In this study, 38 barley and wheat varieties grown in Croatia were evaluated over three consecutive years to assess the effects of cereal type, growing season, and malting on total phenolic content (TPC) and antioxidant activity (AOA). Wheat and barley are essential small grains because of their importance in human and animal nutrition and their wide adaptability. Barley showed significantly higher TPC (1.37 mg Gallic Acid Equivalent (GAE)/g dw) and AOA (63.34%) compared to wheat varieties (TPC 0.85 mg GAE/g dw; AOA 20.09%), with hull-less barley having the highest values (TPC 1.48 mg GAE/g dw; AOA 66.76%). The growing year significantly affected the accumulation of phenolic compounds, with 2019 yielding the highest TPC (1.46 mg GAE/g dw) and AOA (59.85%). Malting enhanced TPC and AOA, particularly in spring barley, with the most pronounced increase recorded in 2017. Statistical analyses demonstrated clear grouping of samples based on growing year and malting status, with hull-less barley varieties forming stable clusters. Hull-less barley varieties such as GZ-192 have emerged as a valuable source of natural antioxidants with potential application in health-promoting food products.

## 1. Introduction

Wheat (*Triticum* spp.) and barley (*Hordeum vulgare* L.) are traditional food crops that are produced across continents in significant quantities and over large areas. They belong to the group of small grain cereals cultivated because of their importance for human and livestock nutrition. In their natural form, cereal grains provide complex carbohydrates, protein, and dietary fibre, lipids rich in unsaturated fatty acids, and essential vitamins and minerals [1,2,3,4]. Due to their variability and agronomic adaptability, small grain cereals make up a large part of crop production in the EU. According to the latest data relating to the production of major cereals, 125.9 million tonnes of wheat and spelt were harvested in 2023. This is 0.8 million tonnes lower than in 2022 (a decrease of 0.6%), mainly due to lower yields, as the harvested area remained largely unchanged at 21.9 million hectares (–0.1%). Barley was particularly affected, falling by 8.9% to 47.4 million tonnes, 4.6 million tonnes less than the previous year. For comparison, during the research period, the average three-year production of wheat and spelt in the EU amounted to 125.1 million tonnes, with the lowest production achieved in 2018 (115.6 million tonnes) and the highest in 2019 (131.8 million tonnes). In the same experimental period, the quantities of barley produced at the EU level were 51.6, 50.1, and 55.6 million tonnes for 2017, 2018, and 2019, respectively. Currently, wheat and barley account for almost two-thirds of total cereal production in the EU [5]. However, cereal production is becoming more challenging due to increasingly frequent adverse weather conditions, such as droughts and heavy autumn rainfall, which can reduce the quantity and quality of the crop. Soil conditions and crop rotation, water availability, high production costs, reduction in land allocated for cereals, and declining yields are also important factors that can affect cereal production [6]. The development of agricultural production is closely related to the domestication of barley and wheat. Today, wheat (*Triticum aestivum* L.) is a widely cultivated crop, the third cereal in total world production after maize and rice, and second only to rice in human nutrition [7,8]. Due to its good adaptability to various climate conditions and long storability, wheat is used in preparation of a diverse variety of foods, thus representing nutritional basis of human diet on a global scale [9,10]. The cultivation of barley is mainly focused on the production of animal feed and malt used in brewing and whiskey distilling manufacture. A smaller amount of barley is used for human consumption [11,12].

A diet rich in whole grains is associated with a lower incidence of cardiovascular and gastrointestinal disorders, certain types of cancer, type-2 diabetes, and obesity. These benefits are largely attributed to the presence of bioactive compounds naturally occurring in the grain matrix. Of particular interest among these are phenolic compounds, which are primarily secondary metabolites produced by plants. They play a vital role in plant defence mechanisms through their antimicrobial and antioxidant activities, helping plants cope with various biotic and abiotic stresses such as pathogen attacks, ultraviolet radiation, drought, and extreme temperatures. In cereal grains, the most abundant classes of phenolic compounds include phenolic acids, flavonoids, and tannins, including proanthocyanidins and hydrolysable tannins, which collectively contribute to the functional properties of the grain and potential health benefits in human nutrition [2,13,14,15,16,17]. These health benefits are largely attributed to the antioxidant and anti-inflammatory properties of phenolic compounds. Antioxidants help maintain oxidation-reduction homeostasis, which is vital for human health. Antioxidant activities include scavenging reactive oxygen species or inhibiting their formation, scavenging electrophiles, and metal chelating activity. And it is widely believed that it is healthier to obtain natural antioxidants by consuming foods rich in bioactive compounds rather than through various substitute preparations [3,17,18]. Given their content of phenolic compounds, along with their availability and widespread use in the diet, barley and wheat have attracted significant interest. Among small grain cereals, hull-less barley stands out as a particularly rich source of total phenolics and flavonoids, as well as other bioactive constituents such as β-glucans and tocopherols [2,19,20].

Barley is a widely used grain for the production of malt due to its favourable proportion of starch and protein and the presence of husk, which results in effective malting. The malting process causes significant changes in the physical and chemical structure of the grain. It is a germination process carried out under technologically controlled conditions where enzymes are activated and synthesized de novo with the aim of converting storage macromolecules into the substrates needed to process malt into beer [21,22,23,24,25]. Some studies have used malting to investigate the potential benefits of the process on polyphenol content and antioxidant capacity in cereal grains. According to Vingrys et al. [26], a significant increase in TPC in all wheat, barley, and sorghum grain samples was attributed to the effect of malting. Radical scavenging activity was also significantly higher in malted samples compared to unmalted samples, except for malted wheat compared to wheat grain. In a study conducted on spring and winter barley varieties, all antioxidant activity values measured by five commonly used tests (2,2-diphenyl-1-picrylhydrazyl (DPPH) radical scavenging activity, TPC, Cupric reducing antioxidant capacity (CUPRAC), Ferric reducing antioxidant power (FRAP), Azino-bis (3-ethylbenzothiazoline-6-sulfonic acid) diammonium salt (ABTS) radical scavenging activity) were higher in malt samples than in the corresponding grain samples [27]. The genotype of malting barley was found to have significant, though small, impact on the TPC and DPPH and ABTS^+^ activities, reducing power and iron (II) chelating activity, but not on O_2_^−^ scavenging activity, whereas growth environment had a minor influence. Malt production conditions, including germination time and malt processing by conventional kiln drying also had a significant impact on TPC and most antioxidant parameters [28]. Different stages of the malting process greatly affect the total phenolic content and overall antioxidant capacity of barley grains and malt. Ha et al. [29] reported that TPC and DPPH radical scavenging potential increased with germination time during the first 48 h and then began to decline when germination was extended to 67 h. Germination for 24 h resulted in a significant increase in the antioxidant properties of different barley varieties. Further increasing the germination duration to 48 h increased the total flavonoids and total phenolic content, DPPH radical scavenging activity, metal chelating activity, and ABTS^+^ scavenging activity [30]. Some differences were detected when comparing barley grains before malting, green malt, and dried malt. As observed by Özcan et al. [31], the highest total phenolic content, antioxidant activity, free phenolic compound content, and carotenoid content were observed in green malt compared to barley grains and finished malt. Quan et al. [32] reported in their study that steeping resulted in a significant reduction in free phenolic content. With continued germination, there was an increase in phenolic content during the first two days, followed by a decrease again after 96 h of germination compared to raw grains.

Examining genotypic variation within plant populations is essential, as environmental factors are known to significantly influence biochemical, morphological, and phenotypic traits, while leaving the underlying genome unchanged. Building on this premise, our study aimed to detect such discrepancies within the analysed parameters, in order to differentiate subgroups within the broader population of barley and wheat genotypes. One notable biochemical change observed during malting is the increase in phenolic compound content, which is primarily attributed to enzyme activation during the steeping and germination phases. Germination therefore provides a natural way to increase the availability of bioactive compounds. In this context, identifying cereal genotypes enriched with health-promoting micronutrients offers valuable potential for improving the nutritional profile of cereal-based diets. The main objective of the present study was to evaluate the total phenolic content and antioxidant activity of selected barley and wheat varieties. Other objectives of this study were (1) to compare TPC and AOA between different small grain cereal species, (2) to assess the influence of growing year on TPC and AOA, and (3) to determine the TPC and AOA in barley varieties after the malting process has been completed.

## 2. Results

### 2.1. Evaluation of Total Phenolic Content and Antioxidant Activity of Extracts from Different Cereal Varieties

A total of 38 varieties were tested across the three trial seasons: 10 genotypes of winter hulled barley, 8 genotypes of winter hull-less barley, 10 genotypes of spring hulled barley, and 10 genotypes of winter bread wheat. The genotypes are listed in Table 1.

**Figure 1:**
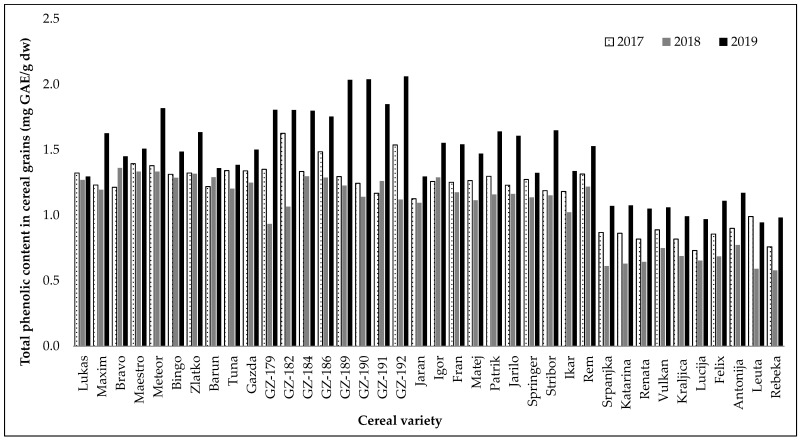
Total phenolic content in grains determined for 38 different cereal varieties grown in the period 2017–2019. The data shown are the mean values of laboratory measurements in triplicate.

After each growing season, the collected cereal grain samples were ground into whole grain flour and analysed for total phenolic content and antioxidant activity. The results of total phenolic content in extracts obtained from flour of different cereal genotypes are presented in Figure 1. TPC is expressed as milligrams of gallic acid equivalents per gram of dry weight (mg GAE/g dw). The content of total phenolics varied from 0.58 mg GAE/g dw (wheat variety Rebeka in 2018) to 2.06 mg GAE/g dw (hull-less barley GZ-192 in 2019) (Table S1). A significant difference (*p* < 0.0001) in mean TPC values was observed between total barley samples (1.37 mg GAE/g dw), included in this study, and total wheat samples (0.85 mg GAE/g dw), respectively (Table 2). Among the groups of different barley types, significantly (*p* < 0.0001) higher total phenolic content was found in hull-less varieties (1.48 mg GAE/g dw, mean value over three years), compared to hulled varieties, both winter and spring (1.33 mg GAE/g dw, mean value over three years). At the same time, there was no statistically significant (*p* > 0.05) difference in the content of total phenolics between the mean values obtained for winter and spring hulled barley (1.37 mg GAE/g dw and 1.30 mg GAE/g dw, respectively, mean values).

When looking at results for all three years of the field trials, it is evident that the all-cereal varieties showed the lowest content of total phenolic compounds in 2018 (Figure 1). The results of TPC in winter hulled barley samples ranged from 1.20 mg GAE/g dw (varieties Maxim and Tuna in 2018) to 1.82 mg GAE/g dw (variety Meteor in 2019) (Table S1). Varieties of hull-less barley generally showed higher values for TPC than the other cereals included in this study, with the maximum reached in 2019 for the variety GZ-192 (2.06 mg GAE/g dw). However, in the second year of field trials (2018), some varieties of hull-less barley exhibited visibly lower values of the total phenolic content, such as GZ-179 and GZ-182 (0.93 and 1.07 mg GAE/g dw, respectively), being among the lowest results achieved for all barley samples. The total phenolic content in the examined spring barley varieties ranged from 1.02 mg GAE/g dw for variety Ikar in 2018 to 1.65 mg GAE/g dw for variety Stribor in 2019. The lowest contents of total phenolic compounds were obtained in wheat samples methanol extract. Results revealed that total phenolic contents of wheat samples were in the range between 0.58 mg GAE/g dw (Rebeka in 2018) and 1.17 mg GAE/g dw (Antonija in 2019).

**Figure 2:**
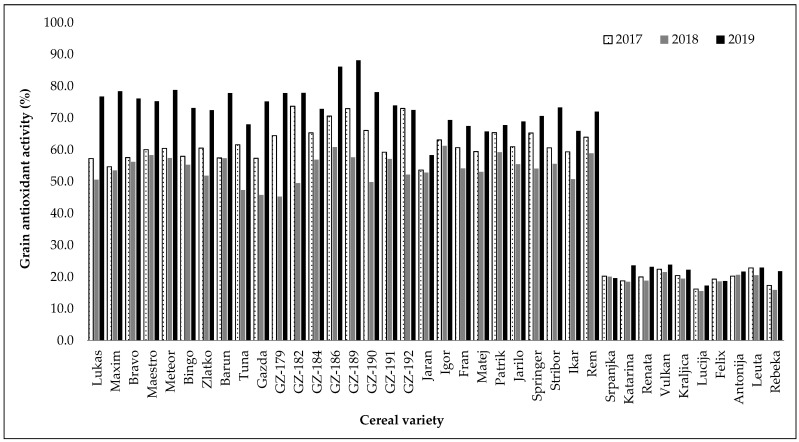
Grain antioxidant activity determined for 38 different cereal varieties grown in 2017–2019. The data shown are the mean values of laboratory measurements in triplicate.

The antioxidant activity results obtained through the DPPH assay followed a trend comparable to that observed for total phenolic content (Figure 1 and Figure 2). Hull-less barley methanol extracts (66.76%, mean value) had the highest significant (*p* < 0.05) DPPH radical scavenging followed by winter hulled barley (62.36%), spring hulled barley (61.58%), and wheat (20.09%) (Table 2). There was no significant (*p* > 0.5) difference in DPPH antioxidant activity between winter and spring hulled barley varieties. The research showed that wheat varieties (20.09%) had significantly (*p* < 0.0001) lower antioxidant activity compared to the overall result of barley varieties (63.34%).

Considering the hull-less barley varieties, the lowest DPPH radical scavenging activity was found in variety GZ-179 (45.27%) from 2018 and the highest in variety GZ-189 (88.17%) from 2019 (Table S1). Among the covered varieties of barley, including both winter and spring types, the lowest values of antioxidant activity were also measured in samples from 2018, and the highest values in samples from 2019. Winter hulled barley variety Gazda (45.81%) had the lowest and Meteor (78.80%) the highest antioxidant activity. Among spring barleys, the lowest (50.74%) antioxidant activity was found in variety Ikar, while the highest (73.31%) value of antioxidant activity was observed in variety Stribor. Wheat varieties Lucija and Rebeka showed the lowest antioxidant activities, reaching 15.57% and 15.87%, respectively, in the second (2018) year of field trials, whereas the varieties Katarina and Vulkan showed the highest DPPH radical scavenging activity, reaching 23.63% and 23.84%, respectively, in the third (2019) year of field trials (Table S1).

### 2.2. Effect of Cereal Type on Total Phenolic Content and Antioxidant Activity

**Figure 3:**
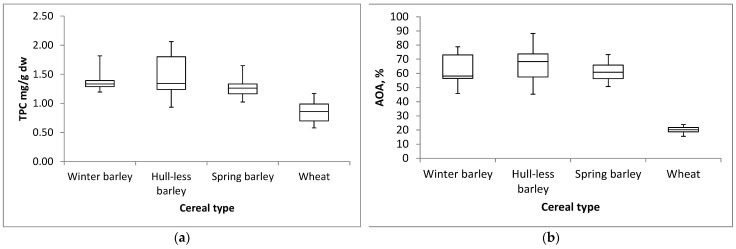
Box and whiskers plots of the relative distribution (min., max., 25% and 75% fractiles, and median (central horizontal line) values) of (**a**) total phenolic content (TPC) and (**b**) antioxidant activity (AOA, assessed by DPPH radical scavenging assay) in grains for four different cereal types (winter barley: *n* = 30; hull-less barley: *n* = 24; spring barley: *n* = 30; wheat: *n* = 30).

A three-year analysis of TPC in methanol extracts of cereals showed that there was a large variability within the results obtained for samples of different varieties of barley and wheat (Figure 3a). Although winter hulled barley and winter hull-less barley varieties had similar median values of TPC values (1.33 and 1.34 mg GAE/g dw, respectively), at the same time hull-less barley showed the highest variability with minimum TPC value of 0.93 mg GAE/g dw and maximum TPC value of 2.06 mg GAE/g dw. Other cereals appeared more stable in terms of content of total phenolics across years having similar differences between maximum and minimum values. In general, winter and spring barley varieties showed comparable contents of total phenolics considering all of the three-year measurements. Differently, wheat samples accumulated a lower content of total phenolics (0.86 mg GAE/g dw, median value) when compared to barley samples (Figure 3a).

Variation in DPPH radical scavenging assessments distribution between different cereal varieties is showed in Figure 3b. The highest (68.35%) median value of antioxidant activity was accessed for hull-less barley and the lowest (20.17%) for wheat samples. There was also the lowest variability between the antioxidant activity results obtained for wheat varieties with a minimum level of 15.57% and a maximum level of 23.84%. On the other hand, the highest variability was recorded within the results obtained for hull-less barley, where the minimum value of antioxidant activity was 45.27% and the maximum value was 88.17%. Interestingly, distributional characteristics of antioxidant activity levels for winter hulled and winter hull-less genotypes were somewhat comparable in terms of minimum and inter-quartile range values. On the contrary, the apparent differences between the plots are due to the higher median and maximum obtained for hull-less barley. On the other hand, spring barley varieties showed relatively high and stable levels of antioxidant activity. Considering the observed levels of antioxidant activity in each type of cereal from all three years, wheat varieties showed the lowest, but stable distribution of antioxidant activity values.

### 2.3. Variation in Total Phenolic Contents and Antioxidant Activities Between Growing Years

**Figure 4:**
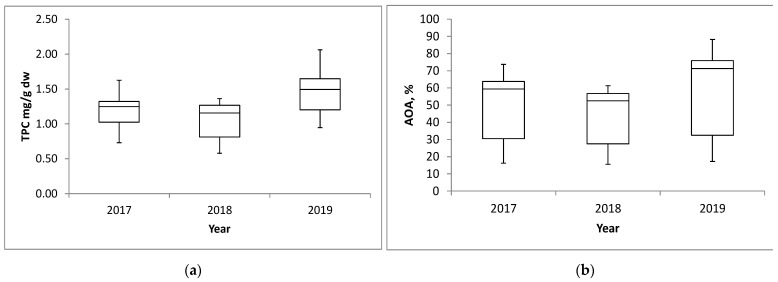
Box and whiskers plots of the relative distribution (min., max., 25% and 75% fractiles, and median (central horizontal line) values) of (**a**) total phenolic content (TPC) and (**b**) antioxidant activity (AOA, assessed by DPPH radical scavenging assay) in grains over three growing years (*n* = 38).

In order to assess the possible influence of the year of cultivation, TPC results were compared between the three years of cultivation, taking into account the data related to each type of cereal as a whole (Figure 4a). Growing years had a significant effect (*p* < 0.001) on the accumulation of total phenolics in selected cereal grains. In the 2019 growing season, the total mean TPC level reached its highest value, amounting to 1.46 mg GAE/g dw. On the contrary, in the previous growing year 2018, all analysed varieties contained significantly (*p* < 0.001) less TPC (1.06 mg GAE/g dw, median value) than in 2019 and 2017. Total TPC results in methanol extracts of cereals from samples collected during the first year of field trials ranged from 0.73 mg GAE/g dw to 1.63 mg GAE/g dw. However, the median value in 2017 was 1.25 mg GAE/g dw, somewhere between the following years 2018 and 2019. As previously stated, the lowest accumulated total phenol contents were in 2018, with a minimum level of 0.58 mg GAE/g dw and a maximum level achieved that year of 1.36 mg GAE/g dw, taking into account all analysed varieties. In 2019, the minimum measured value for TPC was 0.95 mg GAE/g dw, and the maximum level was 2.06 mg GAE/g dw, which are values very similar to the results achieved for hull-less barley, looking at data from all three years as a whole (TPC values for hull-less barley ranged from 0.93 mg GAE/g dw to 2.06 mg GAE/g dw). These results emphasize that environmental factors have a strong influence on TPC accumulation. The largest difference in absolute value of 1.11 mg GAE/g dw between the maximum and minimum values achieved was measured in 2019, but the greatest variability in TPC accumulation with a difference between the maximum and minimum values of about 2.35 times was in 2018.

Evaluation of antioxidant activity across three growing seasons revealed that environmental conditions had a significant impact on grain antioxidant activity with the same sign of influence as was the case with total phenolic content. The lowest median value (52.51%) of antioxidant activity was achieved in 2018, and the highest (71.73%) was achieved in 2019 (Figure 4b). The analysed grain samples from all three harvest years showed comparable minimum levels of antioxidant activity, 16.22%, 15.57%, and 17.26% for 2017, 2018, and 2019, respectively. The minimum values during all three growing years are actually the results achieved by the Lucija wheat variety during the research years. Regarding the maximum levels of antioxidant activity, there were visible differences between different years. The highest maximum level of antioxidant activity of 88.17% was shown by the hull-less barley variety GZ-189 in 2019, and the lowest maximum level of antioxidant activity (61.25%) was shown by the spring barley variety Igor in 2018.

### 2.4. Effect of Malting on Total Phenolic Content and Antioxidant Activity of Barley Varieties

Many previous studies have shown well that barley grain has a good potential for TPC accumulation as well as a good antioxidant potential. In addition to its primary purpose as livestock feed, barley is, among other grains, most widely used for the production of malt in the brewing industry. Malting involves germination under controlled conditions, which results in structural and biochemical modification of the grain that contributes to its nutritional value. The germination process has been shown to have a positive effect on increasing the content of biologically active compounds in grains and thus represents a natural way of contributing to the antioxidant activity of cereal grains [23,31,33]. In this study, barley samples that had previously undergone the malting process were evaluated for total phenolic content and antioxidant activity of the resulting malted grain.

As previously stated, hull-less barley varieties contained significantly more TPC and showed higher DPPH radical scavenging activity than hulled varieties (Table 3). The results demonstrated that malted samples had higher phenolic contents when compared to grain samples. The malting process involves enzyme activity and probably changes in extractability of samples, which can be the reason for the higher phenolic content of malt [31]. The calculated average increase in total phenolic content between malt and grain samples was highest for spring barley varieties and amounted to 30.98%. Average increases in malt phenolic content for winter hulled and hull-less barley varieties were 23.83% and 27.16%, respectively. Antioxidant activities based on the scavenging of DPPH radical were also found to be higher in malt samples than in grain samples. The lowest percentage increase (0.42%) in DPPH radical scavenging activity was determined for the Gazda variety, a winter hulled barley in 2018. On the other hand, the Tuna variety grown in the first year of 2017 showed the highest rate of increase (35.32%) in antioxidant activity. Considering the calculated average three-year values of the degree of increase in TPC and antioxidant activity for each genotype, the results presented in Table 3 show that there were no statistically significant differences between genotypes within the group belonging to the same barley type. On the other side, the statistically significant effect of the growth year was observed, except for the group of hull-less barley genotypes.

### 2.5. Principal Component Analysis (PCA) and Unweighted Pair Group Method Analysis (UPGMA)

**Figure 5:**
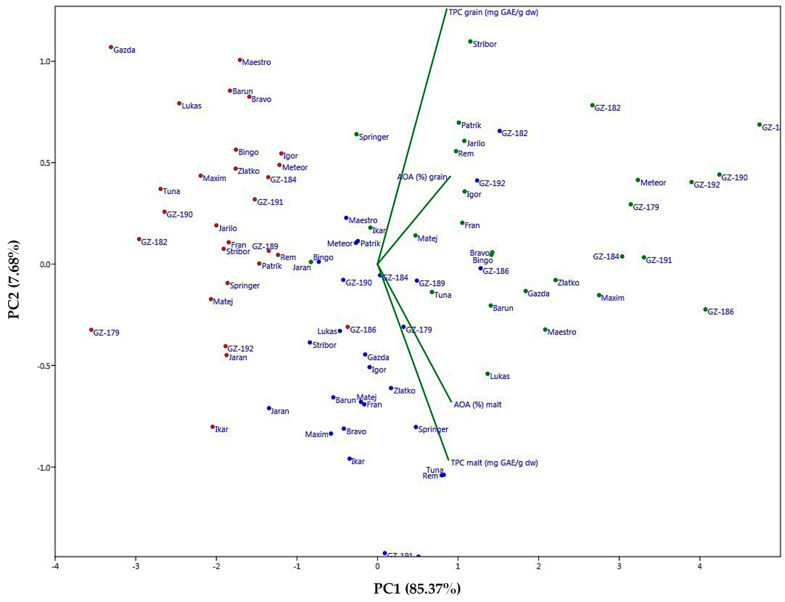
PCA analysis for TPC and AOA in grain and malt (2017—green, 2018—red, 2019—blue).

To examine further the data sets of the observed barley population obtained for the increase and/or modifications achieved through the malting process, the TPC and AOA values were processed and presented via PCA analysis that clearly showed groupings of the barley material over several levels. The first two principal components (PC1 and PC2) explained 93.05% of variance. As can be seen in Figure 5, the first obvious visualized level contains a division of samples by growth year, where 2017 and 2019 show similarities and overlap to some extent through a positive correlation within the first quadrant mainly and through the third and fourth to a lesser extent. The year 2018 largely occupied the second quadrant, clearly setting it apart further from other growth seasons. Bi-plots for grain and malt TPC and AOA values were divided in a way where vectors combined themselves together according to either grain or malt values, which is analogous to the numerical data observation presented in Table 3.

**Figure 6:**
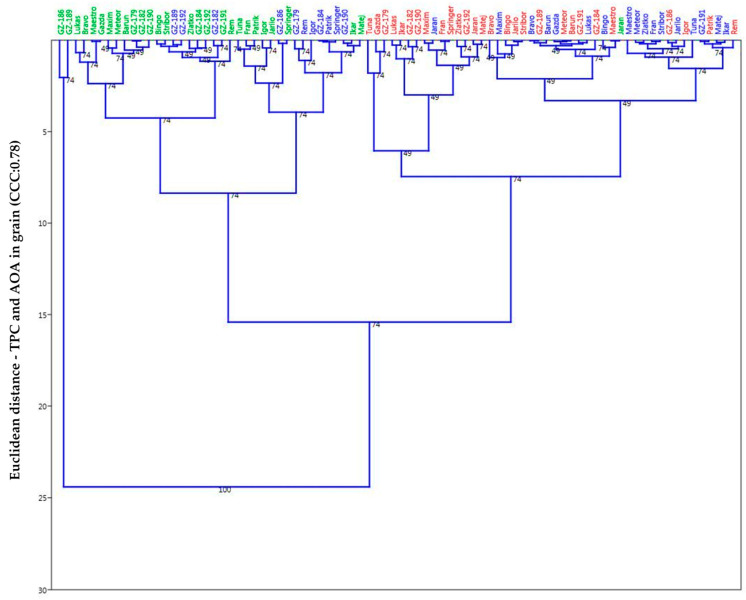
UPGMA dendrogram for TPC and AOA in grain (2017—green, 2018—red, 2019—blue; Cophenetic correlation coefficient: 0.78).

**Figure 7:**
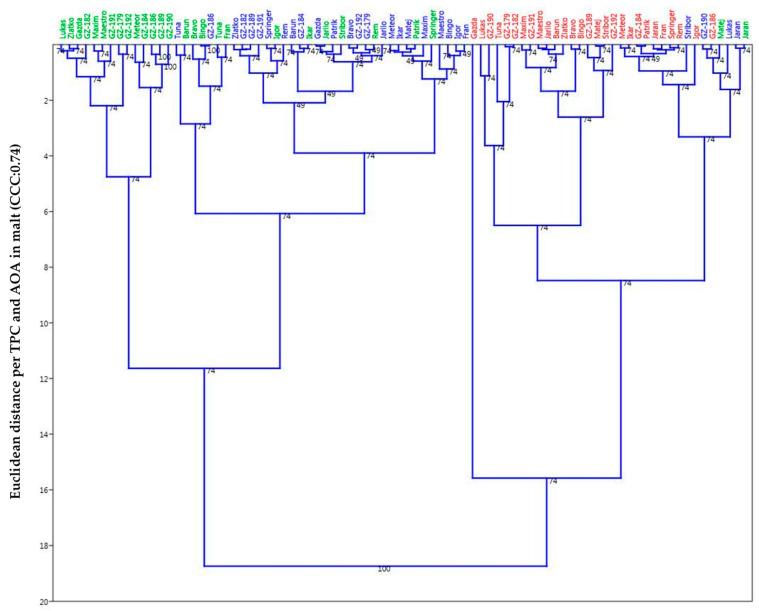
UPGMA dendrogram for TPC and AOA in malt (2017—green, 2018—red, 2019—blue; Cophenetic correlation coefficient = 0.74).

Both TPC and AOA for grain had a very high correlation just as TPC and AOA for malt as well. By this analytic and the vectors shown, one can see with certainty that the malting as a process has the utmost leverage in shaping the groupings of the observed population. The significance of year of growth (season) represents the second level of groupings that possibly evokes and highlights the genotype*environment interaction as a significant contributing factor, and tertiary would be the genotype or the sample itself where similar genotypes (hull-less, winter, and spring barley types) showed proclivity to group together based on the calculated similarity matrix (Euclidean distance) according to TPC and AOA and presented through the UPGMA dendrograms (Figure 6 and Figure 7). If we consider the clusters that have formed, then it can be seen that throughout all the images shown, a certain group or cluster of hull-less samples sticks together almost as a rule and is mostly combined with hulled winter types, so it can be considered that they form the most stable clusters compared to the others.

According to the UPGMA dendrograms and subsequent cluster analysis the malted barley (Figure 7) showed more clear distinction between years/seasons of growth (2017 and 2019 overlap), even more than with unmalted samples (Figure 6), which would point to the conclusion of higher or more active potential physiological activity or similar malting properties that are probably dependant on the genotype*environment interaction or even towards the agro-ecological characteristics of the growing season as was mentioned previously. Cophenetic correlation coefficients on both dendrograms (grain: 0.78; malt: 0.74) have remained relatively high, meaning the modelling for pairwise groupings has merit with minimal distortions towards the original base values. It is furthermore shown that graphic display, as facilitated by this exploratory data analysis, can be utilized in order to evaluate genotype*environmental interactions by considering the position and movements of the individual objects (genotypes in this instance) in the score plots. Thus, in contrast to the classical analysis of variance, the samples can be individually evaluated and the corresponding loadings can be used to validate the genetic and environmental effect of a given sample through quality perspective. Therefore, when observing the formed groups/clusters, one must also take into consideration the length of the growing season, where for example spring barley has a significantly shorter season than winter types; however, this momentum could be examined further and more from the angle of the agricultural sciences in future research.

### 2.6. Bivariate Linear Model Methodology

**Figure 8:**
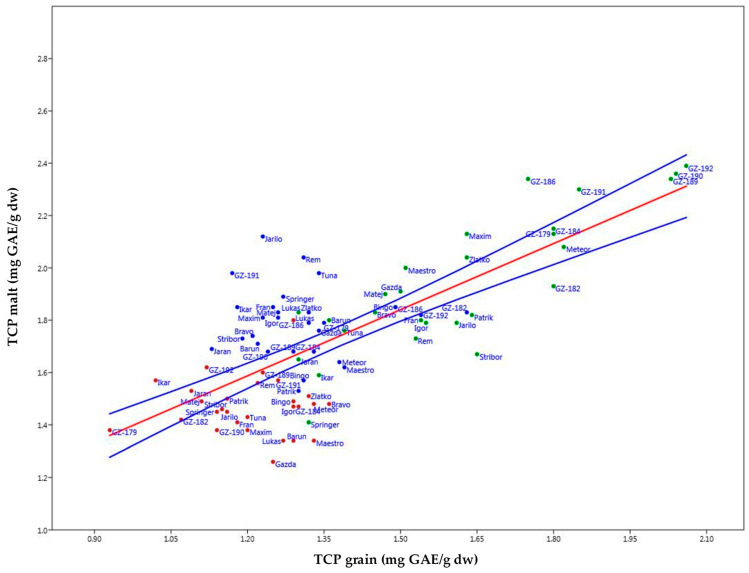
Barley samples levels of TPC in malt plotted against TPC in grain displayed as an increase of parameter influenced by malting across three growing seasons (2017—green, 2018—red, 2019—blue; blue lines represent the 95% confidence interval).

**Figure 9:**
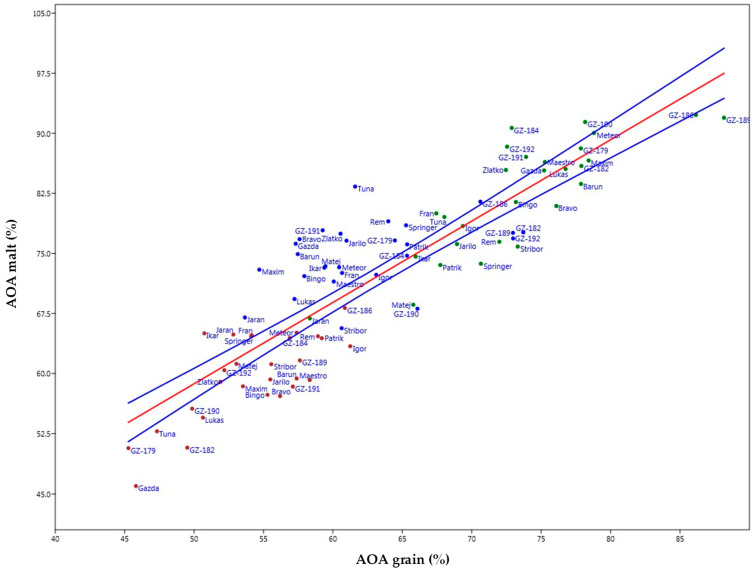
Barley samples levels of AOA in malt plotted against AOA in grain displayed as an increase of parameter influenced by malting across three growing seasons (2017—green, 2018—red, 2019—blue; blue lines represent the 95% confidence interval).

Plotting of the graphs within a bivariate linear model (Figure 8 for TPC and Figure 9 for AOA) showed adequate levels of r^2^ values—0.56 and 0.78, respectively. In Figure 8, the increase in TPC after malting can be examined where, within the 95% confidence interval, the hull-less barley varieties were again fixed at the high end of the predictive spectrum in all growing seasons except for 2018, especially for 2017: GZ-192, GZ-186, GZ-184; 2018: GZ-189, Rem (spring, hulled), Patrik (spring, hulled); 2019: GZ-190, GZ-192, GZ-189.

Situational awareness moves slightly within the AOA parameter (Figure 9) through the seasons highlighting the varieties for 2017: GZ-186, GZ-184 (winter hull-less), and Igor (spring, hulled); 2018: GZ-186, Meteor (winter, hulled), GZ-184; 2019: GZ-186, Meteor (winter, hulled), GZ-179. Again, what gets corroborated in the overall distinction of the increase in values among samples, as was the case with TPC, is the growing season of 2019 that shows the highest results of the analysed parameter, insinuating that it was a season of relatively high stressor levels overall. Deeper analysis according to varietal distinctions show a peculiarity with the variety Meteor, which showed congruence and stability in the overall increase of TPC and AOA remaining firmly within the spectrum of relatively highly reactive but stable genotypes, similar to hull-less samples. This momentum could indicate a potential resistance trait factor of the variety that could be examined through further research. Again, some deeper insight into the production season in accordance with agro-ecological conditions could give possible reasons for why such a case occurred, especially if certain abiotic and biotic stressors have been present at the time of growth. Conclusively, both parameters (TPC and AOA) may be used as proxy-values for post hoc determination of the level of impact of stressors that occurred during the production season for barley in general terms.

## 3. Discussion

Polyphenols are the most abundant antioxidants in the human diet and are of increasing interest as a characteristic that contributes to the nutritional quality of plants. The DPPH radical scavenging assay and the Folin–Ciocalteu method are widely used methods to evaluate the antioxidant capacity and total phenolic content of various plant species. Antioxidant capacity refers to the overall ability of a plant or food to capture free radicals and prevent their damaging effects. DPPH is a stable free radical that absorbs light at 515–517 nm to give a deep purple colour. When applied in assays, the radical is converted to a reduced form by accepting either a hydrogen atom or an electron from an antioxidant species (or reducing agents), appearing yellow in colour when scavenged. Phenolic compounds are natural antioxidants that have been found to be effective in the prevention and treatment of numerous chronic and degenerative diseases. There is currently a growing scientific interest in screening and quantifying phenolic compounds due to their contribution to the antioxidant potential of various plants, such as fruits, herbs, vegetables, and cereals [34,35].

The observed TPC values in selected genotypes align with previously reported data on antioxidant activity and polyphenol content in barley and wheat grains [13,18,36]. A recent study underlined that a significant difference (*p* < 0.001) in total phenolics was observed between methanol extracts of purple barley (78 ± 6.1 mg GAE/100 g dw), purple wheat (35 ± 0.4 mg GAE/100 g dw), and blue wheat (40 ± 2.1 mg GAE/100 g dw) [3]. Previously, several studies have confirmed these natural variations in bioactive compounds contents in cereal grains. Significant differences in antioxidant activity and phytochemical content were observed among the studied types of small grains. Hull-less barley had the highest content of total phenolics and flavonoids, polyvinylpolypyrrolidone (PVPP)-bound phenolics and contained flavan-3-ols, which were not found in other species. Also, the best antioxidant activities assessed as radical scavenging activity with DPPH reagent and the highest reducing power were measured in hull-less barley samples [2]. Free and bound phenolic compounds were examined from four varieties of dehulled highland barley from China. The content of free phenolics ranged from 167.9 ± 12.1 to 282.0 ± 5.5 mg GAE/100 g grain (DW). The main portion of phenolic compounds was found in the bound fraction, varying from 166.0 ± 5.0 to 199.0 ± 4.1 mg GAE/100 g grain (DW) [37].

Examining the differences in antioxidant properties and total phenolic content between einkorn wheat, barley, and bread wheat, Fogarasi et al. [38] observed that einkorn and barley samples exhibited considerable antioxidant activities determined by DPPH and ABTS radical scavenging activities. Among all samples, barley showed the highest antioxidant potential and polyphenol content, while einkorn samples had higher polyphenol content than other wheat samples. Results of another study indicated that hull-less barley can be a good source of phenolic compounds as TPC values ranged from 143.6 mg GAE/100 g to 262.1 mg GAE/100 g [39].

In another study, the antioxidant capacity of grain extracts from different cereal cultivars grown in Latvia and Norway was analysed. Three hull-less barley varieties/lines were tested along with two hulled barley varieties, and three hull-less oat varieties and two hulled oat varieties were also included in the study. Comparing both types of cereals, it was found that barley varieties had significantly higher TPC and ABTS scavenging activity compared to oat varieties, while differences in DPPH scavenging activity between oats and barley were not significant. The highest content of TPC was detected in hull-less barley samples. It was reported that TPC values for barley ranged from 351 mg GAE/100 g dw (hulled barley variety Rubiola from Latvia) to 460 mg GAE/100 g dw (hull-less barley line “GN 03386” from Norway). Also, the results show that the influence of variety on TPC, DPPH, and ABTS scavenging activities is more significant than the type of grain—hull-less or hulled [40]. Recent research showed that barley genotype, growth environment, and their interactions significantly impact the total phenolic content and antioxidant activities [28]. Our results confirm previous findings that hull-less barley accumulates higher TPC. However, the three-year field data further reveal that this stability persists despite significant environmental variation.

Grain polyphenols are often identified as the main contributors to the antioxidant activity of many different cereals. Comparison of antioxidant activity and polyphenol content showed a positive linear correlation (R^2^ = 0.95) for cereal grains, while a weaker (R^2^ = 0.47) correlation coefficient was achieved between total polyphenol content and antioxidant activity for the corresponding malt samples. The action of hydrolytic enzymes during malting results in the release of certain bound polyphenolic compounds with potential antioxidant properties, but these results suggest that not all compounds have antioxidant activities after release [41]. Quantitative profiling of total phenolic content and DPPH radical scavenging activity in sprouts and grasses of maize, wheat, and barley revealed a strong positive correlation between TPC and AOA values [18].

During the malting process, barley grains undergo significant physical and biochemical modifications. With an initial increase in moisture content, germination begins, allowing the embryo to grow under controlled conditions. The germination process allows the seed to develop enzymes that are involved in breaking down the endosperm structure and mobilizing starch and protein reserves and converting them into substrates needed for brewing beer [22,42,43]. In addition to the hydrolysis of the main grain constituents, the malting process has a major impact on changes in the phenolic profile and antioxidant potential. Changes in total phenolic content are mainly due to enzymatic degradation of cell wall structures and better release of bound phenolic compounds, which could lead to higher TPC extraction levels [24,32,44,45].

As reported by Ha et al. [29], the content of total phenolics increased during the first two days of germination, from 1.06 mg/g in non-germinated barley grain to the highest level of 3.37 mg/g after the first 48 h of germination, and then began to decline, reaching 1.98 mg/g after 67 h [29]. Bangar et al. [30] observed a significant increase (up to 35.3%) in TPC in barley grains that were allowed to germinate within 24 h. Analysis of the total phenolic content after extending the germination process to 48 h showed a further increase in TPC among all barley cultivars, ranging between 3898 μg GAE/g and 4604 μg GAE/g. An increase in DPPH radical scavenging activity was also observed in flour samples obtained from barley grains germinated for 24 h by 21.2 to 50.2%. Further increase in germination time to 48 h led to an increase in DPPH activity by 47.4 to 77.2%, with values ranging from 31.1 to 39.4% [30]. A study on the qualitative and quantitative levels of selected phenolic components of barley during the malting process showed that the content of free phenolics decreased significantly during the steeping phase, but then progressively increased during the germination phase. The authors emphasize that the reason for the reduction in free phenolic content could be that some phenolic compounds could have migrated into the water during steeping or formed insoluble complexes between phenols and proteins [32]. In another study, antioxidant activity and phenolic content of malt produced on pilot and industrial scales were highest during germination and then decreased with kilning [46].

Germination induces numerous structural and biochemical changes within the grain matrix, including the synthesis of bioactive compounds with strong protective roles against environmental stress, which likely accounts for the early-stage increase in total phenolic content [24,29]. Özcan et al. [31] showed that the germination process significantly affects changes in the content of bioactive compounds. An increase in total phenolic content (from 101.88 mgGAE/100 g to 107.78 mgGAE/100 g) and antioxidant activity (from 66.48% to 67.31%) was observed in the grain after malting, while the highest values were measured for green malt (122.43 mgGAE/100 g and 79.80%). The results showed that green malt contained a higher amount of phenolic compounds than dried malt, accompanied by higher antioxidant activity [31]. These higher levels in green malt are associated with the activation of hydrolytic enzymes during the steeping and germination phases and changes in extraction capacity [24,31,47].

In our previous study, we showed that TPC values were higher in all kilned malt samples than in barley grains [33]. The percentage increase in TPC between unmalted and malted samples ranged from 3.69% to 40.18% for winter hulled varieties, and from 22.71% to 32.08% for winter hull-less barley varieties. Consequently, DPPH inhibition values were also higher in malt extracts, with an increase rate from 0.36% to 10.66% for winter hulled barley varieties, and from 7.37% to 18.03% for winter hull-less barley varieties. According to a study by Ondrejovič et al. [41], the TPC of different cereal varieties increased during the malting process to 1.2–3.4 times higher values than in non-germinated cereals. Antioxidant capacity values expressed in mg of Trolox equivalents (TEAC) per gram of plant material also increased during malting. Compared to grain, the antioxidant capacity of malt samples produced from wheat and oat varieties was 2.0 to 3.2 higher, while the spring barley variety had comparable antioxidant capacity values between malt and grain samples.

## 4. Materials and Methods

### 4.1. Plant Material and Field Trials

A total of 38 varieties of small grain cereals created at the Agricultural Institute Osijek were grown during three years of research (2017–2019). The field trials were conducted in the eastern part of Croatia, Osijek area (45°33′20″ N, 18°41′40″ E, 94 m above sea level). The plant material included twenty hulled barley varieties (*Hordeum vulgare* L.), eight hull-less or naked barley varieties (*Hordeum vulgare* var. *nudum*), and ten wheat varieties (*Triticum aestivum* L.). The genotypes used in this study were chosen to represent both hulled and naked barleys, with distinction of winter and spring seasonal type within hulled barley varieties. Ten bread wheat varieties were evaluated together with barley in order to gain insight into the existence of possible differences in phenolic content and antioxidant activity between cereal types. Soil type at the growing site is eutric cambisol with a slightly alkaline reaction (pH_KCl_ of 7.17), 2.02% of organic matter content, 29.50 mg of P_2_O_5_, and 34.90 mg of K_2_O 100 g^−1^ of soil [48]. Plants were grown under natural rain-fed conditions. Monthly precipitation amounts and mean monthly temperatures are given in Table S2. Average data were obtained from the Croatian Meteorological and Hydrological Service. Winter crops were sown during fall (1st until 15th of October for each year) and the spring types during the period of 15th until 30th of February, with 450 grains/m^2^ on 7.56 m^2^ plots. Non-limiting levels of nutrients were applied prior to sowing of the winter types with 50 kg ha^−1^ of UREA (46% of N), with subsequent application of 400 kg ha^−1^ of NPK fertilizer (formulation 7:20:30) as a starter and with additional fertilizing just before the dawn emergence stage with 70 kg ha^−1^ of KAN (27% of N).

### 4.2. Micro-Malting

In this study, in addition to the analysis of barley and wheat grains, barley malt samples prepared in laboratory of Agricultural Institute Osijek as part of the regular annual malt quality assessment were also analysed. Barley grains from all years of cultivation were malted under the same conditions using an Automated Joe White Malting Systems Micro-Malting Unit (Joe White Malting Systems, Melbourne, VIC, Australia). Samples of each type of barley were malted in a separate run. Before the malting process, the barley samples were sieved on a shaker with three sieves with slits 2.8, 2.5, and 2.2 mm wide, and only grains larger than 2.5 mm were used for further analysis. The malting procedure was carried out as described in the previous research [49]. To begin the germination process, grain samples were left to stand for 37 h in steeping containers to achieve a target moisture content of approximately 44–46%. The steeping protocol was as follows: 5 h at 16 °C, submerged; 12 h rest in air with 100% air flow at 17 °C; 6 h at 17 °C, submerged; 12 h rest in air with 100% air flow at 18 °C; 2 h at 17 °C, submerged. Germination was maintained at 17 °C for 96 h with 75% air flow and 1.5 rotations every 2 h. Green malts were subjected to 18 h integrated kilning program—6 h at 60 °C; 3 h at 65 °C; 2 h at 68 °C; 2 h at 70 °C; 2 h at 80 °C; 2 h at 83 °C and 1 h at 85 °C—to produce approximately 5–7% moisture malt. After removing the dried rootlets, the malt samples were stored in wide-mouth plastic jars with a suitable stopper and lid at room temperature. Like grain samples, malt samples were analysed for TPC and DPPH radical scavenging activity.

### 4.3. Analysis of Total Phenolic Content and Antioxidant Activity

Whole grain barley and wheat flours as well as barley malt samples were prepared by grinding in a cyclone hammer mill (Laboratory Mill 3100; Perten Instruments AB, Huddinge, Sweden) to pass through a 0.8 mm sieve, and then the powder was stored at 4 °C until use. Extracts were prepared using 2 g of ground barley and wheat grain (or malt) sample mixed with 5 mL of acidified methanol (HCl/methanol, 1:100, *v*/*v*). The mixture was stirred for 2 min by vortexing. Ultrasound extraction lasted for 1 h at room temperature using a Sonorex Digitec ultrasonic bath (RK510 H Model; Bandelin Electronic, Berlin, Germany). Finally, extracts were centrifuged at 9000 rpm for 5 min at 4 °C (Universal 320R; Hettich GmbH & Co. KG, Tuttlingen, Germany). Supernatants were collected, and two rounds of re-extraction were performed with 5 and 2 mL of acidified methanol, respectively. Supernatants were pooled and stored in the dark at –20 °C until analyses of total phenolic content and antioxidant activity.

#### 4.3.1. Determination of Total Phenolic Content

The total phenolic content of grain and malt was determined in three replicates according to the Folin–Ciocalteu method, described by Singleton and Rossi [50], with certain modifications. For TPC determination, 0.1 mL of methanol extract was taken to react with 0.1 mL of Foli–Ciocalteu phenol reagent (1:1) and 1.5 mL of distilled water. The mixture was left to homogenise for 5 min, and then 0.3 mL of 20% Na_2_CO_3_ solution was added. The mixture was shaken thoroughly and incubated for 60 min at room temperature in the dark. Absorbance readings were performed at 765 nm using a UV–Vis spectrophotometer (Specord 200; Analytik Jena GmbH, Jena, Germany) against methanol as a blank. The total phenolic contents are expressed as gallic acid equivalents per gram dry weight (mg GAE/g dw).

#### 4.3.2. Analysis of Antioxidant Activity

DPPH radical scavenging assay was used to evaluate the antioxidant activity of barley, wheat, and malted barley grain extracts. This test enables the identification of antioxidants in cereal grain extract using the 1,1-diphenyl-2-picryl hydrazyl (DPPH) free radical in methanol solution. In this study, the analysis was performed using a modified version of the method explained by Brand-Williams et al. [51]. In a test tube, 1 mL of a 0.5 mmol/L methanol solution of DPPH and 2 mL of methanol were combined with 0.2 mL grain extract. After combining, the test tubes were incubated in complete darkness for 30 min. The absorbance (*A*) of the solution was measured against a methanol blank at 517 nm using a UV-VIS spectrophotometer (Specord 200 Double Beam UV/Vis Spectrophotometer; Analytik Jena GmbH, Jena, Germany). The following equation was used to calculate the inhibition of free radical DPPH in percent (%):

(1) $$\%\;of\;antioxidant\;activity=\left[1-(A_{of\;{sample}_{t=30}}/A_{of\;{controle}_{t=0}}\right]\times 100,$$

where $A_{of\;{sample}_{t=30}}$ of the sample was the absorbance of the tested sample solutions, and $A_{of\;{controle}_{t=0}}$ of the control was the absorbance where the sample was replaced with methanol.

### 4.4. Statistical Analysis

Descriptive statistics and analysis of variance were performed using Statistica 14.2 (TIBCO Software Inc., Palo Alto, CA, USA). Tukey’s post hoc honestly significant difference (HSD) test was used for pairwise comparisons, and significant differences were declared at *p* < 0.05. Box-and-whisker plots were created using Microsoft Excel (Microsoft Office).

#### 4.4.1. Principal Component Analysis (PCA)

PCA determines a set of orthogonal vectors that optimally capture the variability of the data in order of the variance explained in the loading vector directions determined through an eigenvalue decomposition of the covariance matrix [52]. The resulting principal components (PCs) are linear combinations of the original set of variables. The objective of PCA is to identify patterns in data and express their similarities and differences through their correlations [53]. The level of significance for variance component estimates was calculated by non-parametric permutation procedures (10,000 permutations) and bootstrapped.

#### 4.4.2. Unweighted Pair Group Method Analysis (UPGMA)

The method of unweighted average binding among clusters, better known as UPGMA, has been used most frequently in ecology and systematics [54] and in numerical taxonomy [55]. UPGMA is treated as a clustering technique that uses the (unweighted) arithmetic averages of the measures of dissimilarity, thus avoiding characterizing the dissimilarity by extreme values (minimum and maximum) between the considered genotypes. As a general rule, the construction of the dendrogram is established by the genotype(s) of greatest similarity. However, in this case, the Euclidean distance [56] has been calculated and supplied by:

(2) $$D_{ij}=\frac{{\left[\sum_{i}^{p}{\left(x_{im}-x_{jm}\right)}^{2}\right]}^{\frac{1}{2}}}{4M},$$

where $D_{ij}$ represents the Euclidean distance between values of individuals *x*_i_ and *x*_j_, for the variable *x*_m_, that is the average across three seasons. *M* is the overall number of traits observed (TPC and AOA), with 4 M as a representative of the normalizing constant.

#### 4.4.3. Results from Bivariate Linear Model Analysis

In order to explain more in depth the influence of the malting process towards the accumulation and overall results of the increase in TPC and AOA, a simple bivariate linear regression has been performed. Years or growing seasons as a distinct variable were not included in the overall calculus for extraction of the general mean because of the already explained differences and for the overall focus on effects that malting has as a representative in a fixed model with fixed effects of all years combined. Therefore, a linear bivariate model following the approach of Mejza et al. [57] was used in a simplistic formulation for analysis of the data for the differential genotypic calculation (Model 1):

(3) $$Y^{\prime }=a+b*X$$

where *Y*′ is the predicted score, *a* is the intercept or a fixed effects including trait means and the trait by genotype effects, *b* is the slope or the predicted unit increase (whether TPC or AOA), and *X* is the observed value. The level of significance for variance component estimates was calculated by non-parametric permutation procedures (2000 permutations).

All of the calculations were performed with PAST v. 4.03 software package [58].

## 5. Conclusions

To conclude from this study, a considerable variation in TPC and antioxidant activity calculated as DPPH radical scavenging was determined between different cereal types. Barley, especially hull-less genotypes, contained higher TPC and exhibited higher AOA than wheat varieties. The effect of the malting process resulted in an increase in TPC and AOA in all barley samples analysed. On average, the highest increase in total phenolic content between malt and grain samples was found for spring barley varieties and amounted to 30.98%. To compare, the increase in DPPH radical scavenging activity ranged from 12.17% to 15.66%, depending on barley type. The results showed that the growing season significantly affects TPC and antioxidant activity levels. The highest amounts of TPC and the highest antioxidant potentials were observed during the 2019 growing season, while the lowest were recorded in 2018. The observed differences can be attributed to environmental conditions and the occurrence of other stress factors during the growing seasons. Throughout the growing seasons, hull-less barley varieties consistently exhibited the highest stability and most abundant content of phenolic compounds. By using PCA and UPGMA analysis, the varieties were clearly differentiated according to the type of barley, malting process carried out, and growing season. The most stable clusters were invariably formed by hull-less barley varieties, which were often grouped together with hulled winter varieties.

These findings highlight that specific barley types, particularly hull-less forms, represent valuable sources of natural antioxidants and that malting can enhance their nutritional quality. The results can also contribute to the selection of high-potential genotypes. Certainly, the way in which genotype and growth environment influence TPC and AOA, as well as the possibilities of applying specific grain processing procedures to improve the antioxidant status of grains, could be the subject of further research.

## Figures and Tables

**Table 1:** List of 38 genotypes of small grain cereals grown for three years.

Cereal Type	Genotype	Year of Registration	Field Trials
Winter hulled barley	Lukas	2010	2016–2017 2017–2018 2018–2019
	Maxim	2009	
	Bravo	2009	
	Maestro	2010	
	Meteor	-	
	Bingo	2005	
	Zlatko	1999	
	Barun	2001	
	Tuna	2014	
	Gazda	2007	
Winter hull-less barley	GZ-179	Breeding line	2016–2017 2017–2018 2018–2019
	GZ-182	Breeding line	
	GZ-184	Breeding line	
	GZ-186	Breeding line	
	GZ-189	Breeding line	
	GZ-190	Breeding line	
	GZ-191	Breeding line	
	GZ-192	Breeding line	
Spring hulled barley	Jaran	1983	2017 2018 2019
	Igor	1998	
	Fran	2002	
	Matej	2002	
	Patrik	2004	
	Jarilo	-	
	Springer	2009	
	Stribor	2009	
	Ikar	2010	
	Rem	2007	
Wheat	Srpanjka	1989	2016–2017 2017–2018 2018–2019
	Katarina	2006	
	Renata	2006	
	Vulkan	2009	
	Kraljica	2010	
	Lucija	2001	
	Felix	2007	
	Antonija	2011	
	Leuta	2011	
	Rebeka	2011	

**Table 2:** Mean and range of total phenolic content and antioxidant activity as affected by cereal type and growing year.

	TPC ^1^ (mg GAE/g dw)		DPPH ^2^ (% Inhibition)	
Cereal Type	Mean ^3^	SD ^6^	Mean	SD
Winter hulled barley	1.37 b	±0.14	62.36 b	±10.03
Winter hull-less barley	1.48 a	±0.34	66.76 a	±11.62
Spring hulled barley	1.30 b	±0.17	61.58 b	±6.26
Winter wheat	0.85 c	±0.17	20.09 c	±2.28
Year	Mean ^4^	SD	Mean	SD
2017	1.18 b	±0.23	51.07 b	±19.51
2018	1.06 c	±0.26	44.95 c	±16.18
2019	1.46 a	±0.32	59.84 a	±23.85
Cereal Crop	Mean ^5^	SD	Mean	SD
Barley	1.37 a	±0.23	63.34 a	±9.54
Wheat	0.85 b	±0.17	20.09 b	±2.28

^1^ Total phenolic content. ^2^ DPPH radical scavenging activity. ^3^ Data are mean values of three-year results (winter hulled barley: *n* = 30; winter hull-less barley: *n* = 24; spring hulled barley: *n* = 30; winter wheat: *n* = 30). ^4^ The data are the mean values of the results determined for each year (*n* = 38). ^5^ The data are the mean values of the results determined for overall barley (*n* = 84) and wheat samples (*n* = 30). ^6^ Standard deviation. Mean values followed by different letters within a column are significantly different at the *p* < 0.05 level (Tukey’s HSD post hoc test).

**Table 3:** Total phenolic contents and antioxidant activity of barley grain and malt extracts.

Variety/ Year	TPC ^1^ (mg GAE/g dw)		TPC Incr. ^2^ (%)	DPPH ^3^ (% Inhibition)		DPPH Incr. (%)
	Grain	Malt		Grain	Malt	
Winter hulled barley						
Lukas	1.30 ^4a^ ± 0.03	1.65 ^a^ ± 0.27	27.24 ^a^ ± 19.36	61.54 ^a^ ± 13.58	69.78 ^a^ ± 15.53	13.39 ^a^ ± 6.95
Maxim	1.35 ^a^ ± 0.24	1.77 ^a^ ± 0.38	31.22 ^a^ ± 15.92	62.21 ^a^ ± 14.05	72.66 ^a^ ± 14.10	17.66 ^a^ ± 13.66
Bravo	1.34 ^a^ ± 0.12	1.68 ^a^ ± 0.18	25.99 ^a^ ± 17.42	63.29 ^a^ ± 11.11	71.63 ^a^ ± 12.68	13.80 ^a^ ± 17.04
Maestro	1.41 ^a^ ± 0.09	1.65 ^a^ ± 0.33	16.46 ^a^ ± 15.95	64.55 ^a^ ± 9.32	72.36 ^a^ ± 13.63	11.77 ^a^ ± 9.17
Meteor	1.51 ^a^ ± 0.27	1.73 ^a^ ± 0.31	14.82 ^a^ ± 3.72	65.55 ^a^ ± 11.58	76.14 ^a^ ± 12.72	16.30 ^a^ ± 4.27
Bingo	1.36 ^a^ ± 0.11	1.64 ^a^ ± 0.19	20.15 ^a^ ± 4.30	62.13 ^a^ ± 9.66	70.31 ^a^ ± 12.15	13.17 ^a^ ± 10.54
Zlatko	1.42 ^a^ ± 0.18	1.79 ^a^ ± 0.27	25.94 ^a^ ± 11.78	61.64 ^a^ ± 10.32	73.95 ^a^ ± 13.57	19.81 ^a^ ± 7.35
Barun	1.29 ^a^ ± 0.07	1.61 ^a^ ± 0.24	25.19 ^a^ ± 19.17	64.24 ^a^ ± 11.80	72.66 ^a^ ± 12.30	13.76 ^a^ ± 14.50
Tuna	1.31 ^a^ ± 0.10	1.72 ^a^ ± 0.28	31.26 ^a^ ± 14.78	58.98 ^a^ ± 10.60	71.90 ^a^ ± 16.65	21.28 ^a^ ± 12.46
Gazda	1.36 ^a^ ± 0.13	1.64 ^a^ ± 0.34	19.98 ^a^ ± 16.39	59.45 ^a^ ± 14.81	69.19 ^a^ ± 20.60	15.63 ^a^ ± 16.37
2017	1.31 ^5b^ ± 0.06	1.74 ^b^ ± 0.12	33.75 ^a^ ± 11.88	58.49 ^b^ ± 2.10	74.79 ^b^ ± 3.94	27.92 ^a^ ± 6.03
2018	1.28 ^b^ ± 0.06	1.41 ^c^ ± 0.09	9.58 ^b^ ± 6.65	53.38 ^c^ ± 4.37	56.89 ^c^ ± 5.02	6.62 ^c^ ± 5.10
2019	1.51 ^a^ ± 0.15	1.92 ^a^ ± 0.13	28.15 ^a^ ± 7.00	75.21 ^a^ ± 3.28	84.50 ^a^ ± 3.14	12.43 ^b^ ± 3.78
Winter hull-less barley						
GZ-179	1.36 ^a^ ± 0.44	1.77 ^a^ ± 0.38	32.57 ^a^ ± 14.52	62.53 ^a^ ± 16.38	71.81 ^a^ ± 19.15	14.69 ^a^ ± 3.65
GZ-182	1.50 ^a^ ± 0.38	1.73 ^a^ ± 0.27	17.66 ^a^ ± 13.97	67.04 ^a^ ± 15.32	71.44 ^a^ ± 18.36	6.06 ^a^ ± 3.92
GZ-184	1.48 ^a^ ± 0.28	1.77 ^a^ ± 0.35	19.53 ^a^ ± 6.32	65.04 ^a^ ± 8.00	76.61 ^a^ ± 13.21	17.34 ^a^ ± 6.15
GZ-186	1.51 ^a^ ± 0.23	2.00 ^a^ ± 0.30	32.54 ^a^ ± 7.53	72.54 ^a^ ± 12.75	80.65 ^a^ ± 12.07	11.51 ^a^ ± 4.14
GZ-189	1.52 ^a^ ± 0.45	1.88 ^a^ ± 0.41	25.21 ^a^ ± 8.66	72.92 ^a^ ± 15.28	77.05 ^a^ ± 15.16	5.85 ^a^ ± 1.41
GZ-190	1.47 ^a^ ± 0.49	1.81 ^a^ ± 0.50	23.90 ^a^ ± 10.08	64.70 ^a^ ± 14.20	71.71 ^a^ ± 18.17	10.51 ^a^ ± 7.02
GZ-191	1.43 ^a^ ± 0.37	1.95 ^a^ ± 0.36	39.71 ^a^ ± 26.15	63.43 ^a^ ± 9.14	74.44 ^a^ ± 14.65	17.14 ^a^ ± 14.62
GZ-192	1.57 ^a^ ± 0.47	1.94 ^a^ ± 0.40	26.17 ^a^ ± 15.86	65.91 ^a^ ± 11.89	75.21 ^a^ ± 14.03	14.29 ^a^ ± 8.32
2017	1.38 ^b^ ± 0.16	1.79 ^b^ ± 0.11	31.04 ^a^ ± 17.31	68.18 ^b^ ± 5.19	76.35 ^b^ ± 3.84	12.49 ^a^ ± 9.57
2018	1.17 ^c^ ± 0.13	1.53 ^c^ ± 0.15	31.82 ^a^ ± 11.82	53.66 ^c^ ± 5.27	58.77 ^c^ ± 6.22	9.56 ^a^ ± 5.05
2019	1.89 ^a^ ± 0.13	2.24 ^a^ ± 0.16	18.63 ^a^ ± 7.71	78.45 ^a^ ± 5.87	89.47 ^a^ ± 2.42	14.47 ^a^ ± 7.03
Spring hulled barley						
Jaran	1.17 ^a^ ± 0.11	1.62 ^a^ ± 0.08	39.01 ^a^ ± 11.34	54.94 ^b^ ± 2.97	66.25 ^a^ ± 1.20	20.77 ^a^ ± 5.41
Igor	1.37 ^a^ ± 0.16	1.69 ^a^ ± 0.19	24.31 ^a^ ± 17.34	64.57 ^a^ ± 4.25	71.39 ^a^ ± 7.55	10.40 ^a^ ± 6.00
Fran	1.32 ^a^ ± 0.19	1.69 ^a^ ± 0.24	28.25 ^a^ ± 17.10	60.75 ^ab^ ± 6.65	72.44 ^a^ ± 7.61	19.28 ^a^ ± 0.58
Matej	1.28 ^a^ ± 0.18	1.74 ^a^ ± 0.22	36.08 ^a^ ± 7.88	59.43 ^ab^ ± 6.37	67.73 ^a^ ± 6.14	14.36 ^a^ ± 9.64
Patrik	1.37 ^a^ ± 0.25	1.62 ^a^ ± 0.18	19.44 ^a^ ± 9.60	64.10 ^a^ ± 4.40	71.37 ^a^ ± 6.16	11.29 ^a^ ± 4.51
Jarilo	1.33 ^a^ ± 0.24	1.79 ^a^ ± 0.33	36.38 ^a^ ± 32.07	61.80 ^a^ ± 6.76	70.67 ^a^ ± 9.89	14.29 ^a^ ± 9.97
Springer	1.24 ^a^ ± 0.10	1.58 ^a^ ± 0.26	27.27 ^a^ ± 20.86	63.35 ^a^ ± 8.44	72.30 ^a^ ± 7.03	14.70 ^a^ ± 9.00
Stribor	1.33 ^a^ ± 0.28	1.62 ^a^ ± 0.14	24.28 ^a^ ± 22.20	63.17 ^a^ ± 9.14	67.56 ^a^ ± 7.52	7.29 ^a^ ± 3.44
Ikar	1.18 ^a^ ± 0.16	1.67 ^a^ ± 0.16	42.85 ^a^ ± 21.02	58.69 ^ab^ ± 7.64	70.95 ^a^ ± 5.18	21.53 ^a^ ± 7.68
Rem	1.35 ^a^ ± 0.16	1.77 ^a^ ± 0.24	31.93 ^a^ ± 21.23	64.97 ^a^ ± 6.59	73.37 ^a^ ± 7.65	13.14 ^a^ ± 9.13
2017	1.24 ^b^ ± 0.06	1.83 ^a^± 0.17	48.21 ^a^ ± 13.66	61.25 ^b^ ± 3.48	73.44 ^a^ ± 4.45	20.00 ^a^ ± 5.47
2018	1.15 ^c^ ± 0.07	1.49 ^b^ ± 0.05	29.76 ^b^ ±10.91	55.53 ^c^ ± 3.30	63.35 ^b^ ± 2.05	14.44 ^ab^ ± 7.90
2019	1.49 ^a^ ± 0.13	1.71 ^a^ ± 0.14	14.97 ^c^ ± 8.63	67.95 ^a^ ± 4.17	74.42 ^a^ ± 4.06	9.67 ^b^ ± 5.15

^1^ Total phenolic content. ^2^ The percentage increases of TPC and DPPH (% inhibition) between malts and barleys are shown in separate columns. ^3^ DPPH radical scavenging activity. ^4^ Means ± standard deviation (3 years, *n* = 3). ^5^ Means ± standard deviation for each cereal type in growing years (winter hulled barley: *n* = 10; winter hull-less barley: *n* = 8; spring hulled barley: *n* = 10) Mean values followed by different letters within a column are significantly different at the *p* < 0.05 level (Tukey’s HSD Post Hoc test).

## Data Availability

Data supporting the findings of this study are available from the corresponding author upon reasonable request.

## Supplementary Materials

The following supporting information can be downloaded at: https://www.mdpi.com/article/10.3390/plants14111664/s1, Table S1: Results of total phenolic content and antioxidant activity over three consecutive years (2017–2019).; Table S2: Precipitation (mm) and temperature (°C) by year and month for the study period.

## Abbreviations

The following abbreviations are used in this manuscript:

ABTS	Azino-bis (3-ethylbenzothiazoline-6-sulfonic acid) diammonium salt
AOA	Antioxidant Activity
CUPRAC	Cupric Reducing Antioxidant Capacity
DPPH	2,2-diphenyl-1-picrylhydrazyl
EU	European Union
FRAP	Ferric Reducing Antioxidant Power
GAE	Gallic Acid Equivalent
PCA	Principal Component Analysis
PVPP	Polyvinylpolypyrrolidone
TEAC	Trolox Equivalent Antioxidant Capacity
TPC	Total Phenolic Content
UPGMA	Unweighted Pair Group Method Analysis
