# Supplementary material for: Genotypic, Environmental, and Processing Effects on Phenolic Content and Antioxidant Activity in Barley and Wheat
Source: Plants (Basel). 2025 May 30;14(11):1664. doi: 10.3390/plants14111664 (PMC12158008; doi:10.3390/plants14111664)
Supplement: Supplementary file 1 [file plants-14-01664-s001.zip › Table S2_Precipitation (mm) and temperature (◦C) by year and month for the study period..pdf]

CLIMATE DATA WERE OBTAINED FROM THE CROATIAN METEOROLOGICAL AND HYDROLOGICAL SERVICE (DHMZ) AT A LOCATION OSIJEK

(45°29' N latitude and 18°48' E longitude)

Croatian Meteorological and Hydrological Service. Weather in Croatia. Available online:
https://meteo.hr/podaci_e.php?section=podaci_vrijeme&prikaz=abc (accessed on 15 March 2025)

YEAR	**SUM OF PRECIPITATION** (mm)												**TOTAL**
	**I**	**II**	**III**	**IV**	**V**	**VI**	**VII**	**VIII**	**IX**	**X**	**XI**	**XII**	**I-XII**
2016	70.6	76.0	58.0	38.1	39.6	164.8	114.2	48.7	37.6	68.0	54.8	0.8	771.2
2017	36.9	52.1	55.7	54.8	50.5	35.7	60.1	24.0	64.6	55.7	37.3	44.0	571.4
2018	59.1	70.9	71.9	25.1	77.6	102.9	89.2	45.6	106.1	13.7	31.9	24.4	718.4
2019	41.7	20.0	11.1	74.7	118.8	105.8	57.3	83.4	61.6	31.3	78.6	48.8	733.1
**LTP* (1991-2000)**	**41.6**	**34.5**	**40.5**	**51.0**	**59.2**	**82**	**66.3**	**61.9**	**51.0**	**55.9**	**61.5**	**48.8**	**654.2**
YEAR	**AVERAGE OF TEMPERATURE** (˚C)												**AVERAGE**
	**I**	**II**	**III**	**IV**	**V**	**VI**	**VII**	**VIII**	**IX**	**X**	**XI**	**XII**	**I-XII**
2016	0.8	6.9	7.5	13.1	16.5	21.0	22.8	20.6	18.1	10.4	6.2	-0.1	12.0
2017	-5.1	3.8	10.0	11.6	19.3	23.4	23.9	24.2	16.5	12.4	6.9	3.5	12.5
2018	4.2	0.9	4.6	17.0	20.6	21.7	22.5	24.4	18.3	14.4	7.6	1.5	13.1
2019	0.3	4.6	9.6	13.2	14.6	23.6	23.3	24.0	18.0	13.5	10.3	4.3	13.3
**LTP* (1991-2000)**	**-0.2**	**1.8**	**6.4**	**11.2**	**16.7**	**19.6**	**21.3**	**20.8**	**16.5**	**11.1**	**5.1**	**1.3**	**11.0**

***LTP** (LONG TERM PERIOD)
